# Large field-of-view intravascular ultrasound offering tomographic perspective online for accurate sizing during transcatheter pulmonary valve replacement

**DOI:** 10.1093/ehjci/jeac075

**Published:** 2022-05-02

**Authors:** Piotr N Rudziński, Łukasz Kalińczuk, Gary S Mintz, Marcin Demkow

**Affiliations:** National Institute of Cardiology, ul. Alpejska 42, 04–628, Warsaw, Poland; National Institute of Cardiology, ul. Alpejska 42, 04–628, Warsaw, Poland; Cardiovascular Research Foundation, New York, USA; National Institute of Cardiology, ul. Alpejska 42, 04–628, Warsaw, Poland

An 18-year-old female with tetralogy of Fallot underwent surgical correction in childhood. Current echocardiography and cardiac magnetic resonance (CMR) showed severe pulmonary insufficiency of implanted 12 mm Contegra valve. Given the CMR insight into the right ventricular outflow tract (RVOT) (*Panel A*), transcatheter pulmonary valve replacement (TPVR) was scheduled. RVOT dimensions assessed in angiography using the sizing balloon corresponded with CMR measurements (*Panel B*). SAPIEN 3 26 mm (Edwards Lifesciences, Irvine, California, USA) with its nominal outer valve frame area of 5.8 mm^2^ was primarily selected. However, a Vision PV035 10 MHz intravascular ultrasound (IVUS; Philips North America Corporation, Andover, MA, USA) was used as a research periprocedural imaging. IVUS showed RVOT with significantly bigger dimensions of 4.9–7.7 cm^2^ and effective pulmonary area derived diameter of 30 mm (*Panel C*). Following pre-stenting with Optimus XXL 38 mm stent deployed on 30 mm BinB (NuMED, Hopkinton, NY, USA), 3 mm larger SAPIEN 3 29 mm (nominal outer frame area of 7.0 cm^2^) was successfully deployed (*Panel D*). Subsequently, IVUS was used to verify valve expansion (*Panel E*, *arrow indicates the transducer location parallel to valve long-axis*). It showed the outer valve area of 7.8 cm^2^ (>100% of the nominal expansion) and visualized leaflet motion in systole and diastole, with a geometric orifice area of 3.1 cm^2^ (*Panel F*). Current guidance of TPVR relies upon the results of pre-procedural imaging and periprocedural angiography with low-pressure balloon inflations. Large field-of-view IVUS offers a unique *online* tomographic perspective with the highest visual resolution for novel optimal TPVR guidance.

**Figure:**
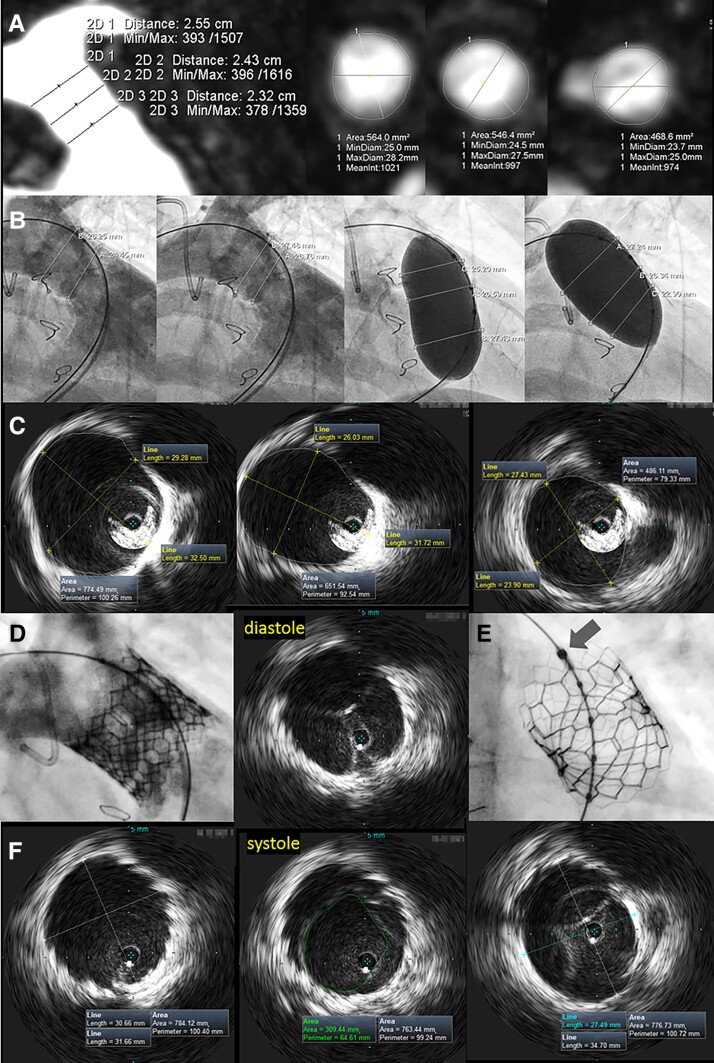


**Conflict of interest:** None declared.

**Funding:** None declared.

